# Histological Assessment of Respiratory Tract and Liver of BALB/c Mice Nebulized with Tocilizumab

**DOI:** 10.3390/pharmaceutics16070862

**Published:** 2024-06-27

**Authors:** Paloma Jimena de Andres, Sergio Ferreiro, Angela Flores, Almudena Garcia, Cesar Henriquez-Camacho

**Affiliations:** 1 Departamento de Medicina y Cirugía Animal, Facultad de Veterinaria de la, Universidad Complutense de Madrid, 28040 Madrid, Spain; pjandres@ucm.es; 2 Unidad de Veterinaria, Radiodiagnóstico y Cirugía Experimental del, Centro de Apoyo Tecnológico de la, Facultad de Ciencias de la Salud de la, Universidad Rey Juan Carlos, 28922 Alcorcon, Spain; sergio.ferreiro@urjc.es; 3 Servicio de Farmacia del Hospital Universitario Rey Juan Carlos, 28993 Mostoles, Spain; afloresj@hospitalreyjuancarlos.es (A.F.); almudena.garcia@hospitalreyjuancarlos.es (A.G.); 4 Servicio de Medicina Interna del Hospital Universitario de Móstoles, 28935 Mostoles, Spain; 5 Facultad de Ciencias de la Salud, Universidad Rey Juan Carlos, 28922 Alcorcon, Spain

**Keywords:** nebulization, animal model, monoclonal antibody, tocilizumab, BALB/c

## Abstract

Pulmonary drug delivery offers a minimally invasive and efficient method for treating lung conditions, leveraging the lungs’ extensive surface area and blood flow for rapid drug absorption. Nebulized therapies aim to deliver drugs directly to the lung tissue. This study investigates the histological impact of nebulized tocilizumab—a monoclonal antibody targeting IL-6, traditionally administered intravenously for rheumatoid arthritis and severe COVID-19—on a murine model. Thirty BALB/c mice were nebulized with tocilizumab (10 mg, 5 mg, and 2.5 mg) and six controls were nebulized with saline solution. They were euthanized 48 h later, and their organs (lungs, nasal mucosa, and liver) were analyzed by a microscopic histological evaluation. The results indicate that all the mice survived the 48 h post-nebulization period without systemic compromise. The macroscopic examination showed no abnormalities, and the histopathological analysis revealed greater lung vascular changes in the control group than in the nebulized animals, which is attributable to the euthanasia with carbon dioxide. Additionally, increased alveolar macrophages were observed in the nebulized groups compared to controls. No significant histological changes were observed in the liver, indicating the safety of nebulized tocilizumab. In conclusion, these findings suggest the potential of nebulized tocilizumab for treating pulmonary inflammation, warranting further research to establish its efficacy and safety in clinical settings.

## 1. Introduction

Pulmonary drug delivery is gaining increasing attention as a minimally invasive and appealing approach to treat numerous medical conditions, particularly those affecting the lungs [1]. Due to the extensive surface area of and abundant blood flow in the lungs, drugs are absorbed more rapidly, leading to enhanced therapeutic effectiveness and a reduced occurrence of side effects [2]. Nebulized lung therapies have been tested in different settings with the purpose of directly reaching the lung tissue to avoid the side effects that occur when systemic treatments, i.e., antibiotics, immunosuppressive drugs, antifungal, etc., are used [3].

Tocilizumab is a recombinant humanized monoclonal antibody that blocks the interleukin 6 (IL-6) receptor [4]. It is approved for the treatment of rheumatoid arthritis and systemic juvenile arthritis [5,6,7]. Tocilizumab has also been used in patients infected with SARS-CoV-2 and resulting in severe coronavirus disease (COVID-19) [8]. Adult Respiratory Distress Syndrome (ARDS) is the most severe complication in patients with severe COVID-19, and inflammatory complications have been associated with a cytokine release syndrome with high mortality [9,10]. For patients with elevated inflammatory markers and pro-inflammatory cytokines, intravenous corticosteroids, baricitinib, and tocilizumab have been used extensively in moderate/severe COVID-19 cases because of its mortality benefit [11,12,13]. The heightened risk of overwhelming infections (bacterial, fungal, and parasitic) is a potential adverse effect of anti-inflammatory treatments [14,15,16]. Minimizing systemic exposure and secondary side effects are critical.

The only route of tocilizumab administration that has been tested in clinical trials for use in humans is intravenous. Apart from inhaled interferon beta-1a [17] and corticosteroids, which have demonstrated positive effects in reducing hospital admissions, mortality rates, and resolving all initial symptoms [18], nebulized treatments for COVID-19 or other inflammatory lung diseases have not undergone testing. Even when intravenous drugs (steroids or tocilizumab) are used during a cytokine storm, some patients develop Acute Respiratory Distress Syndrome (ARDS) with severe lung damage and, consequently, require Intensive Care Unit (ICU) admission for ventilatory assistance [19]. It is necessary to identify new formulations or different approaches to minimize lung damage and sequelae in different scenarios with lung inflammation.

There are no animal models demonstrating the effects of nebulized tocilizumab. The utilization of nebulized monoclonal antibodies targeting interleukin in pulmonary inflammatory ailments lacks empirical data, though numerous inhaled antibodies are currently undergoing initial clinical trials for managing SARS-CoV-2 infection and asthma/COPD [20].

The objective of this study was to observe the histological changes in the respiratory tract and liver of a murine model following treatment with nebulized tocilizumab (liquid formulation developed for intravenous delivery). To that end, a histological study of various organs, including the nasal mucosa, trachea, lungs, and liver, was conducted on the nebulized mice exposed to different doses of tocilizumab.

## 2. Materials and Methods

### 2.1. Animals

BALB/c mice (9 weeks old) were kept in the animal facility at 20–24 °C, 45–55% humidity, and a 12 h/12 h light/dark cycle with food and tap water ad libitum. They were housed in clean-air, viral- and antigen-free facilities with restricted access in the Veterinary Unit at Rey Juan Carlos University. The mice were allowed to acclimate for one week prior to use, and they were used in accordance with an approved protocol. This study was conducted with the approval of the Institutional Animal Care and Use Committee of Rey Juan Carlos University (PROEX 171.4/21).

**Figure 1:**
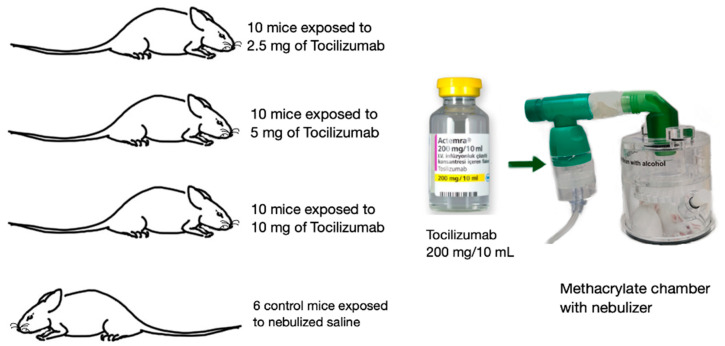
Nebulization of tocilizumab (200 mg/mL) using a polymethyl methacrylate chamber (Emka technologies SAS).

The animals were “whole body” exposed to nebulized tocilizumab or a vehicle (saline) only. During nebulization, the animals were placed in a polymethyl methacrylate (PMMA) chamber (Emka technologies SAS) of 396 cm^3^ with a filter on the top, which was linked to a 17 cm long, nontoxic, corrugated PVC tube, which was attached to the nebulizer (Intersurgical 1453015), with 4 liters per minute of oxygen as the air-jet nebulizer. To avoid contamination of the control group, their nebulization was performed on separate days (Figure 1). The experimental dose determination was reached after a pilot test that included three different doses per mouse (10 mg, 5 mg, and 2.5 mg) of a 20 mg/mL concentration of tocilizumab (Roamctera-Roche Pharma), with sufficient time for complete solution nebulization (mean of 15–20 min). The nebulization was stopped when no solution was observed in the cap of the nebulizer. A control group (3 males and 3 females) exposed to saline (5 mL 0.9% NaCl) and three study groups (5 males and 5 females) were established as follows:

- Control group: 6 control mice exposed to saline (5 mL of saline solution, 0.9% NaCl);
- Group 1: 10 mice exposed to nebulized tocilizumab (2.5 mg in a volume of 5 mL completed with saline) and euthanized after 48 h;
- Group 2: 10 mice exposed to nebulized tocilizumab (5 mg; 5 mL final volume) and euthanized after 48 h;
- Group 3: 10 mice exposed to nebulized tocilizumab (10 mg; 5 mL final volume) and euthanized after 48 h.

Mortality was assessed at 24 and 48 h. If the mice did not die within this time frame, they were euthanized with carbon dioxide (CO_2_), and the organs (lungs, nasal mucosa, and liver) were fixed in 4% buffered paraformaldehyde solution and submitted to a veterinary pathologist (PJdA) who was blinded to the treatment group assignment. The organs were paraffin-embedded, and a single 5 µm section from each sample was acquired and stained with Hematoxylin and Eosin (H&E) for microscopic histological evaluation.

### 2.2. Histopathological Evaluation

The stained slides were evaluated in order to describe the histopathological characteristics. The histological changes evaluated in the respiratory tract were as follows: vacuolization of the epithelial cells, presence of bronchiole detritus, increased height of the bronchiolar epithelium, perivascular infiltration, intra-alveolar macrophage infiltration, thickening of the alveolar septa, observation of intra-alveolar fibrin, intra-alveolar edema, congestion, hemorrhage, and platelet aggregation. The histopathological changes evaluated in the liver samples were the presence of hepatocyte degeneration/necrosis, perivascular inflammation, and focal parenchymatous inflammation.

Each of these changes was semiquantitatively categorized as follows: 0 (absence), 1 (mild), 2 (moderate), and 3 (severe). In addition, a total score for the changes observed in the respiratory tract and liver was established for each animal. Each animal was then categorized based on the histopathological changes observed, and three categories were established as follows: 0 (no histological changes), 1 (score between 1 and 3), 2 (score between 4 and 6), and 3 (score higher than 6).

### 2.3. Statistical Study

The analyses were performed using SPSS 19, and a conventional *p* < 0.05 level was used to define statistical significance.

For the statistical analysis, four groups of animals were compared as follows: the control, group 1, group 2, and group 3. All the histopathological changes were categorical variables with four categories each: absence, mild, moderate, and severe. Similarly, the animal scoring was a categorical variable with four categories: 0, 1, 2, and 3. A Pearson chi-squared statistical analysis was used to test the association among the different categorical variables within the groups.

## 3. Results

All the animals survived for 48 h after nebulization. No signs of systemic compromise were reported prior to euthanasia.

Macroscopically, the trachea, nasal mucosa, and liver of all the groups appeared normal at the time of euthanasia. A similar degree of vascular changes (congestion or hemorrhage) in the lungs was observed in some animals from both the control and experimental groups. The hepatic tissue was macroscopically normal in all the animals in this study.

**Figure 2:**
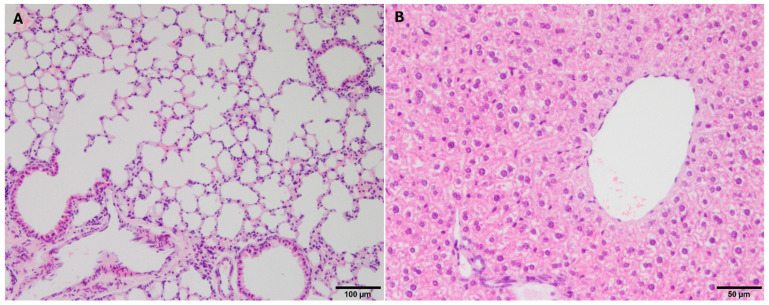
Normal histological architecture in organs from mice nebulized with saline (control group). (**A**) Lung, original magnification (OM) ×10. (**B**) Liver, OM ×20.

**Figure 3:**
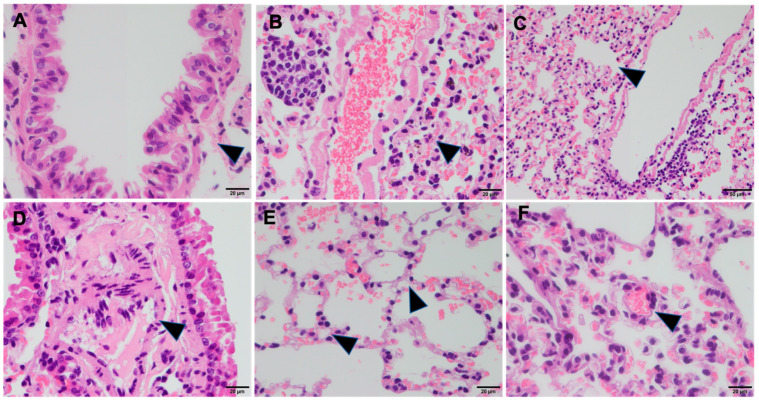
Histopathological alterations observed in lungs of animals treated with nebulized tocilizumab. (**A**) Group 3 shows slight perivascular inflammatory cells infiltration, OM ×20. (**B**) Group 1 shows slight perivascular inflammatory cells infiltration, OM ×40. (**C**) Group 2 shows mild interstitial edema, OM ×40. (**D**) Group 1 shows mild thickening of alveolar septa, OM ×40. (**E**) Group 2 shows vacuole in the epithelium, OM ×40. (**F**) Group 3 shows macrophages in the intra-alveoli, OM ×40.

Microscopically, the organs of the control group animals presented with no signs of inflammation or necrosis, and with normal histological architecture (Figure 2). Mostly mild vascular changes (congestion, edema, and hemorrhage) were observed in the lungs of the animals from all four groups. More animals with lung hemorrhages were found in the control group (83.4%) than in the other groups (40% in group 1, 30% in group 2, and 10% in group 3) (*p* = 0.016). Likewise, a higher percentage of animals with intra-alveolar edema was observed in the control group (50%) compared to the other groups (0% in group 1, 0% in group 2, and 10% in group 3) (*p* = 0.008). The lungs from experimental groups 1, 2, and 3 showed mostly mild histopathological alterations, including slight perivascular inflammatory cell infiltration, mild interstitial edema, mild thickening of the alveolar septa, and scant vacuoles in the epithelium (Figure 3).

None of these evaluations were statistically different than those for the control group (Table 1). However, more macrophages in the alveoli were found in the nebulized mice (group 1, 60%; group 2, 100%; and group 3, 70%) compared to the control group (0%) (*p* < 0.001). No focal tracheal necrosis, change in the height of the bronchiolar epithelium, cell degeneration, or exfoliation were observed.

**Figure 4:**
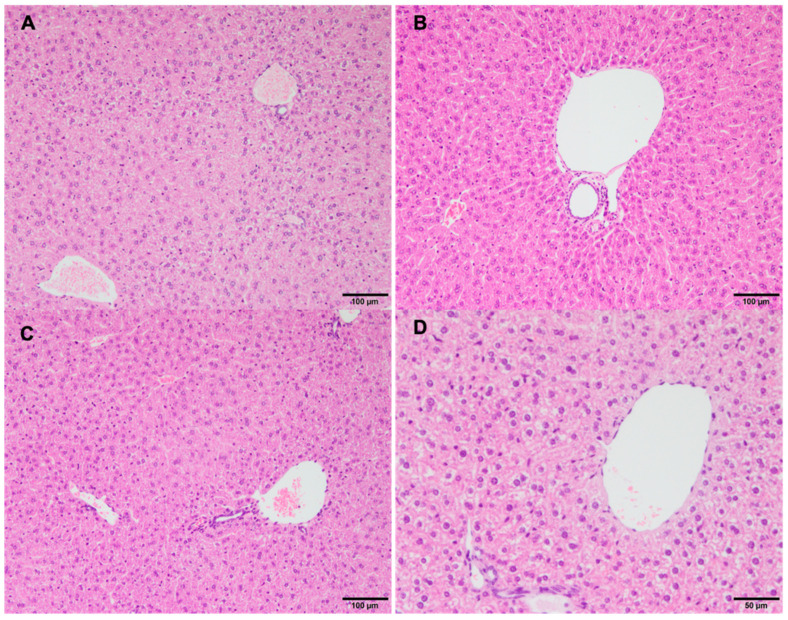
Normal liver histological architecture in animals treated with nebulized tocilizumab. (**A**) Group 1, OM ×10. (**B**) Group 2, OM ×10. (**C**) Group 3. (**D**) Control group, OM ×40.

No histological changes were found in the livers of the animals nebulized with tocilizumab (Figure 4). Lastly, no statistical differences were found for the animal total score among all the groups (*p* = 0.251) (Table 2).

## 4. Discussion

Pulmonary drug delivery has emerged as a promising approach for treating a variety of respiratory conditions due to its ability to target the lungs directly, offering rapid drug absorption and potentially minimizing systemic side effects. This study explores the novel use of nebulized tocilizumab, a monoclonal antibody that inhibits the IL-6 receptor, which has been widely used intravenously for conditions such as rheumatoid arthritis, systemic juvenile arthritis, and severe COVID-19 [7,8]. The severe complications of COVID-19, notably ARDS, often involve a cytokine release syndrome characterized by elevated levels of IL-6, among other markers [9]. Consequently, systemic anti-inflammatory treatments like corticosteroids and tocilizumab have been employed to manage these conditions, albeit with the risk of severe infections as side effects [11].

To date, tocilizumab has only been administered intravenously in clinical settings for COVID-19, with nebulized treatments being largely unexplored, except for inhaled interferon beta-1a and corticosteroids, which have shown efficacy at reducing hospital admissions and mortality in COVID-19 patients [17,18]. Nebulization of bronchodilators is necessary in some COPD/asthma patients with COVID-19, with maximal protection for patient and health workers [21,22,23]. Despite the potential benefits, there is a lack of empirical data on the use of nebulized monoclonal antibodies for treating pulmonary inflammatory diseases. This study aimed to fill this gap by assessing the histopathological changes in the respiratory tract and liver of mice exposed to nebulized tocilizumab.

The results demonstrate that all the mice survived the 48 h post-nebulization period without any signs of systemic compromise. The macroscopic examination revealed no abnormalities in the trachea, nasal mucosa, or liver across all the groups. Interestingly, the control group exhibited a higher incidence of lung hemorrhages and intra-alveolar edema compared to the experimental groups. This suggests that nebulization itself did not exacerbate these conditions. Pulmonary hemorrhage is a common finding in animals euthanized with CO_2_ [24]. Furthermore, it has been observed that the degree of pulmonary hemorrhage that develops depends on the conditions of CO_2_ euthanasia, which seems to have influenced this result [24,25].

The histopathological analysis showed mild vascular changes in and inflammation of the lungs across all the groups, with no significant differences between the control and experimental groups regarding most histological alterations. However, the presence of alveolar macrophages was significantly higher in the nebulized groups compared to the controls. Alveolar macrophages and bronchial/alveolar epithelial cells are the primary cells responsible for the handling of airborne particles in the lungs. Consequently, they produce pro-inflammatory mediators that can trigger both local inflammatory responses in the lungs and systemic inflammatory responses [26,27]. Alveolar macrophages play a pivotal role in the clearance and processing of inhaled environmental microparticles in both the airways and alveolar spaces. The primary function of these cells is to maintain clear air spaces by phagocytosing foreign materials. While processing the engulfed material, alveolar macrophages also function as antigen-presenting cells, thereby activating the adaptive immune response. These innate and adaptive immune responses are mutually interdependent, placing alveolar macrophages at the nexus of the two immune pathways [28]. Thus, this finding indicates an immunomodulatory effect of nebulized tocilizumab, potentially enhancing macrophage activity in the alveolar spaces, which could be beneficial in managing lung inflammation.

Importantly, no histological changes were observed in the livers of the nebulized mice, suggesting that tocilizumab did not induce hepatotoxicity when administered via this route. The absence of tracheal necrosis, bronchiolar epithelial changes, or significant cell degeneration in the nebulized groups further supports the safety of this administration method.

Tocilizumab is a 145 kDa lyophilized protein that is diluted into a liquid hydrophilic formulation. Consequently, it can be employed in mesh (aerosol) or jet (liquid droplet) nebulizing systems for inhalation. Tocilizumab is not a small molecule, as are some of the nebulized compounds that are commonly used in clinical practice (e.g., salbutamol (239.31 Da), budesonide (430.5 Da), and ipratropium bromide with 421.4 Da). Nevertheless, some large molecules, including cyclosporine (1202.6 Da), amphotericin B (924 Da), and colistin (1155.4 Da), have demonstrated clinical efficacy [29,30,31]. Given the observed changes in the nebulized mice, it appears that nebulization was an effective method for delivering the molecule.

Previous studies utilizing nebulized neutralizing antibodies in mice have demonstrated some benefits against viral and bacterial infection [32,33,34]. No antibodies against any interleukin have been tested previously; thus, this represents the inaugural experiment for studying the effects of a nebulized monoclonal antibody in mice based on an intravenous formulation. It was unnecessary to develop a novel formulation for this study, which provides in vivo evidence that cost-effective doses can be employed as nebulized agents to deliver drugs to the lung.

Despite these promising findings, this study’s limitations must be acknowledged. The short observation period (48 h) limits the understanding of the long-term effects and potential delayed toxicities. Additionally, this study was conducted in a controlled environment with specific dosages that may not directly translate to human applications. It is also important to consider that multiple factors are involved during nebulization, including molecular size, solubility, aggregation, temperature, effective clinical doses, and interface exposure. These factors have been discussed in detail in the literature [35,36,37]. Further research involving longer observation periods, varied dosages, and clinical trials in humans will be essential to fully establish the safety and efficacy of nebulized tocilizumab.

This study provides new insights for a better understanding of the effects of nebulized tocilizumab in the respiratory tract and liver of mice. Future studies using models of inflammatory lung diseases are necessary to establish the definitive role of nebulized monoclonal antibodies in the control of local lung inflammation.

## 5. Conclusions

In conclusion, this study provides preliminary evidence supporting the feasibility and safety of nebulized tocilizumab for treating pulmonary inflammation. The findings suggest that nebulization can deliver tocilizumab effectively to the lungs without causing significant systemic or local adverse effects. This opens the possibility for further exploration and potential clinical applications in managing inflammatory lung diseases, including those caused by infections like COVID-19.

## Figures and Tables

**Table 1:** Histopathological changes observed in the respiratory tracts of the animals in this study (control: nebulized with saline; group 1: nebulized with 2.5 mg of tocilizumab; group 2: nebulized with 5 mg of tocilizumab; group 3: nebulized with 10 mg of tocilizumab).

Histological Changes	Degree	Control (N = 6)	Group 1 (N = 10)	Group 2 (N = 10)	Group 3 (N = 10)	*p* Value *
Vacuole on epithelial cells	Absence	6 (100%)	9 (90%)	8 (80%)	9 (90%)	0.665
	Mild	0	1 (10%)	2 (20%)	1 (10%)	
	Moderate	0	0	0	0	
	Severe	0	0	0	0	
Bronchiole detritus	Absence	2 (33.3%)	9 (90%)	10 (100%)	9 (90%)	**0.004**
	Mild	4 (66.6%)	1 (10%)	0	1 (10%)	
	Moderate	0	0	0	0	
	Severe	0	0	0	0	
Perivascular infiltration	Absence	6 (100%)	9 (90%)	8 (80%)	8 (80%)	0.633
	Mild	0	1 (10%)	2 (20%)	2 (20%)	
	Moderate	0	0	0	0	
	Severe	0	0	0	0	
Intra-alveolar macrophage infiltration	Absence	6 (100%)	4 (40%)	0	3 (30%)	**<0.001**
	Mild	0	6 (60%)	10 (100%)	7 (70%)	
	Moderate	0	0	0	0	
	Severe	0	0	0	00	
Thickening of alveolar septa	Absence	1 (16.6%)	5 (50%)	3 (30%)	3 (30%)	0.337
	Mild	4 (66.6%)	5 (50%)	7 (70%)	7 (70%)	
	Moderate	1 (16.6%)	0	0	0	
	Severe	0	0	0	0	
Intra-alveolar fibrin	Absence	3 (50%)	8 (80%)	5 (50%)	6 (60%)	0.505
	Mild	3 (50%)	2 (20%)	5 (50%)	4 (40%)	
	Moderate	0	0	0	0	
	Severe	0	0	0	0	
Intra-alveolar edema	Absence	3 (50%)	10 (100%)	10 (100%)	9 (90%)	**0.008**
	Mild	3 (50%)	0	0	1 (10%)	
	Moderate	0	0	0	0	
	Severe	0	0	0	0	
Vascular congestion	Absence	1 (16.6%)	2 (20%)	0	1 (10%)	0.452
	Mild	3 (50%)	5 (50%)	5 (50%)	8 (80%)	
	Moderate	2 (33.3%)	3 (30%)	5 (50%)	1 (1%)	
	Severe	0	0	0	0	
Hemorrhages	Absence	1 (16.6%)	6 (60%)	7 (70%)	9 (90%)	**0.016**
	Mild	5 (83.4%)	2 (20%)	3 (30%)	1 (10%)	
	Moderate	0	2 (20%)	0	0	
	Severe	0	0	0	0	
Platelet aggregation	Absence	4 (66.6%)	5 (50%)	7 (70%)	3 (30%)	0.288
	Mild	2 (33.3%)	5 (50%)	3 (30%)	7 (70%)	
	Moderate	0	0	0	0	
	Severe	0	0	0	0	

* Numbers in bold denote statistically significant differences.

**Table 2:** Scoring categories for the animals in this study (control: nebulized with saline; group 1: nebulized with 2.5 mg of tocilizumab; group 2: nebulized with 5 mg of tocilizumab; group 3: nebulized with 10 mg of tocilizumab).

Category *	Control (N = 6)	Group 1 (N = 10)	Group 2 (N = 10)	Group 3 (N = 10)	*p* Value
0	0	0	0	0	0.251
1	3 (50%)	9 (90%)	8 (80%)	6 (60%)	
2	3 (50%)	1 (10%)	2 (20%)	4 (40%)	
3	0	0	0	0	

* Categories stablished according to the total score for each animal: 0 (no histological changes), 1 (scoring between 1 and 3), 2 (scoring between 4 and 6), and 3 (scoring higher than 6).

## Data Availability

The dataset used and analyzed in the current study is available from the corresponding author upon reasonable request.
